# Supplementary material for: Aggregation and structural phase transitions of semiflexible polymer bundles: A braided circuit topology approach
Source: iScience. 2024 Jan 26;27(3):108995. doi: 10.1016/j.isci.2024.108995 (PMC10867648; doi:10.1016/j.isci.2024.108995)
Supplement: Document S1. Figures S1 and S2 [file mmc1.pdf]

iScience, Volume *27*

Supplemental information

Aggregation and structural phase transitions of semiflexible polymer bundles: A braided circuit topology approach

Jonas Berx and Alireza Mashaghi

I. DISTRIBUTIONS OF THE CIRCUIT TOPOLOGY MOTIF FRACTIONS

We provide here a detailed breakdown of the distribution of the topological motif fractions as a function of the stiffness κ for $M = 4$ and $N = 10$ (Fig. S1) or $N = 30$ (S2). The colours indicate the probability of a motif having a given fraction on the y-axis. For high stiffness values, all motif fractions stabilise around a fixed distribution.

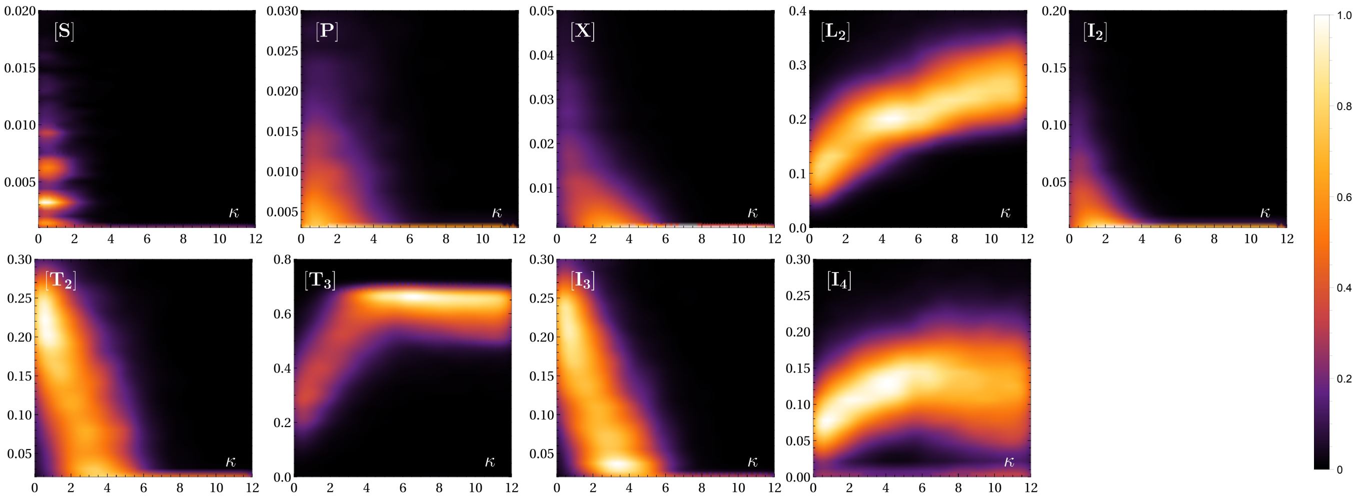

Figure S1: Density plots for the topology fractions of the different CT motifs as a function of stiffness in the system with $N = 10$ monomers per chain, related to Fig. 6.

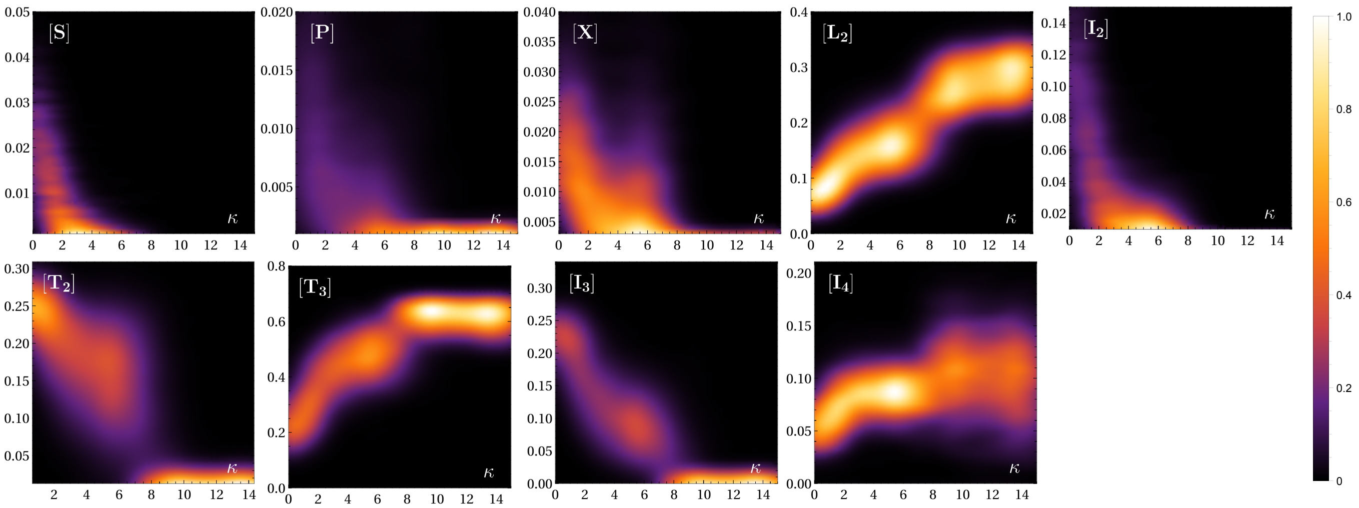

Figure S2: Density plots for the topology fractions of the different CT motifs as a function of stiffness in the system with $N = 30$ monomers per chain, related to Fig. 7.
